# Assessment of wound healing activity in diabetic mice treated with a novel therapeutic combination of selenium nanoparticles and platelets rich plasma

**DOI:** 10.1038/s41598-024-54064-2

**Published:** 2024-03-04

**Authors:** Rania A. Karas, Shaimaa Alexeree, Hassan Elsayed, Yasser A. Attia

**Affiliations:** 1 National Institute of Laser Enhanced Sciences, Cairo University, Giza, 12613 Egypt; 2 Department of Microbial Biotechnology, Biotechnology Research Institute, National Research Centre, Dokki, 12622 Giza Egypt; 3 School of Biotechnology, Badr University in Cairo, Cairo, 11829 Egypt

**Keywords:** Diabetic wound healing, Therapeutic potential, Selenium nanoparticles, Platelet-rich plasma, Nano-combination, Biological techniques, Developmental biology, Evolution, Diseases, Health care, Medical research, Chemistry, Nanoscience and technology

## Abstract

Diabetic wound healing is sluggish, often ending in amputations. This study tested a novel, two-punch therapy in mice—Selenium nanoparticles (Se NPs) and platelet-rich plasma (PRP)—to boost healing. First, a mouse model of diabetes was created. Then, Se NPs were crafted for their impressive antioxidant and antimicrobial powers. PRP, packed with growth factors, was extracted from the mice's blood. Wound healing was tracked for 28 days through photos, scoring tools, and tissue analysis. Se NPs alone spurred healing, and PRP added extra fuel. Furthermore, when used in combination with PRP, the healing process was accelerated due to the higher concentration of growth factors in PRP. Notably, the combination of Se NPs and PRP exhibited a synergistic effect, significantly enhancing wound healing in diabetic mice. These findings hold promise for the treatment of diabetic wounds and have the potential to reduce the need for lower limb amputations associated with diabetic foot ulcers. The innovative combination therapy using Se NPs and PRP shows great potential in expediting the healing process and addressing the challenges of impaired wound healing in individuals with diabetes. This exciting finding suggests this therapy could change diabetic wound management, potentially saving limbs and improving lives.

## Introduction

Diabetes mellitus (DM) is a severe global public health problem. It had been reported that there were 451 million people with diabetes in 2017 worldwide, which would be anticipated to increase to 693 million by 2045 and nearly half of those people are undiagnosed^1^. Diabetes's debilitating impact on wound healing stems from a double whammy of damaged blood vessels (macrovascular and microvascular lesions) and increased susceptibility to infections (bacterial and fungal)^2^. These injuries also put a heavy worldwide social and health burden due to a lack of effective wound healing agents. About 2.5–15 per cent of monthly worldwide health budgets are consumed on diabetes mellitus, and diabetic injuries play a major part^3^. Some studies have reported that sustained high glucose situations in cases with diabetes reduced the skin cell proliferation rate production of collagen which impedes wound healing after skin injury^4,5^. Moreover, pathological microvascular changes, reduced proliferation rate, elevated proinflammatory cytokines, and the absence of growth factors are also associated with delayed wound healing^6,7^.

Healing is a dynamic process that begins as soon as tissue integrity is disrupted. The healing process is divided into four main parts, namely hemostasis, inflammation, proliferation, and remodeling. Activated platelets are important in the first step of tissue repair by promoting neutrophil adhesion, which produces several growth factors such as transforming growth factor-β (TGF-β) and induce monocytes that migrate to the wound bed. Neutrophils play one of the most important roles in infection control by producing a variety of cytokines and growth factors, which aid in the differentiation of circulating monocytes to complete subsets activating or proinflammatory agents (M1) and anti-inflammatory agents (M2)^8^. This facilitates fibroblast activation, angiogenesis of new capillaries, and capillary remodeling during the proliferative phase^9^. The diabetic state consists of an episode of chronic low-grade inflammation characterized by an elevated M1/M2 ratio and a prolonged inflammatory response through increased levels of pro-inflammatory cytokines such as interleukin-1β (IL-1β), interleukin-6 (IL-6) and tumor necrosis factor-α (TNF-α), which, in the long term, impair healing activity after injury^10^. The literature shows an inverse relationship between neutrophil activity and wound healing efficiency, and in diabetes, high neutrophil levels have been found to damage the process of wound healing^11^. Diabetic wounding is also associated with impaired angiogenesis and dysfunction of endothelial progenitor cells, as well as disruption of myoepithelial cells (MECs) secretion^12,13^.

In diabetes, two features seem to be central to explaining the risk of developing damaged skin functions: accelerated skin aging^14,15^ and chronic inflammation, which is particularly prominent pop in diabetic foot ulcers (DFU)^16^. DFU is the most common complication in patients with diabetes; it increases the risk of developing osteomyelitis, which can lead to lower limb amputation^17^. Management of DFU is difficult because it is the result of complex pathophysiology^18,19^. In addition, due to ethical concerns, trials of new therapeutic interventions in humans are limited, delaying the development of effective strategies. Therefore, testing of new approaches to the treatment of DFU must rely, at least in the early stages, on the existence of suitable experimental models^20^.

Recently, the use of nanomaterials in wound healing is growing rapidly due to the special physico-chemical and biological properties of nanoparticles. Having highly specific surfaces has also been found to have potential applications in wound dressings for sustained delivery and release of therapeutic agents. Furthermore, nanomaterials can stimulate a variety of cellular and molecular processes that contribute to the wound microenvironment through antibacterial, anti-inflammatory and angiogenic effects, which can change the environment from non-healing to healing^21^. Overall, the nanoparticles used in wound healing are categorized into metal and metal oxide nanoparticles, peptide nanostructures, polymeric nanostructures, and lipid nanoparticles.

Se NPs are considered multifunctional drugs that have been discovered and shown to be more bioavailable and less toxic^22–24^. Selenium is a necessary trace mineral element involved in almost all human physiological processes but the range from effective to unsafe concentrations of selenium is limited^25^. Se NPs have all the physiological functions of selenium with lower toxicity and higher biological activity. Such characteristics make them ideal to solve the problem of the limited concentration range of selenium^26,27^. Se NPs have vital biological activities such as antioxidant, antibacterial, antiviral, and anti-cancer^28–30^. The antimicrobial studies on Se NPs reveal no bacterial resistance to date. This makes Se NPs ideal candidate for curing infectious and chronic wounds.

Platelet-rich plasma (PRP) is an autologous product rich in growth factors (GFs) which boost tissue regeneration. These GFs include Platelet-derived growth factor (PDGF), transforming growth factor-beta (TGF)-β, vascular endothelial growth factor (VEGF), epidermal growth factor (EGF), fibrinogen, osteocalcin, and insulin-like growth factor (IGF) which are essential for cell proliferation, chemoattractant, and collagen synthesis, macrophages and other growth factors activation and cell metabolism. The increase of these growth factors in PRP stimulates fibroblasts proliferation and collagen type I expression which lead to stimulation of granulation, angiogenesis, cell proliferation, and re-epithelialization^31,32^. In regenerative medicine, PRP is considered a novel strategy that showed tissue repair with better differentiation, recruitment, and proliferation of cells when introduced to wound area because it has a regenerative ability on worn out cells and tissues by tapping into the natural healing processes of the body^33^. Also, it could repair and stimulate the pancreatic cells to produce insulin again. In addition to its potential of reducing blood glucose as well as increasing insulin levels, superoxide dismutase activity (SOD)^34^. Diabetic wounds pose a significant challenge, often proving resistant to healing due to the complex interplay of impaired blood flow, neuropathy, and chronic inflammation. While existing therapies and medications like antibiotics, growth factors, and skin grafting offer some relief, they often have limitations. While the antibiotics are effective against infections, they don't address the underlying diabetic factors hindering healing. While the growth factors are stimulating cell growth and tissue repair, their short lifespan and high cost limit their practicality. Skin grafting often requires donor sites and can suffer from graft rejection.

According to previous research, Se NPs have anti-inflammatory and antioxidant qualities that help speed up diabetic mice's wound healing^28^. Furthermore, because PRP contains growth factors and cytokines that encourage cell proliferation and the production of extracellular matrix molecules, it has been demonstrated to have the ability to aid in wound healing. On the other hand, the possible antidiabetic effects of PRP were not examined before. Thus, the addition of Se NPs to PRP could have been made to boost PRP's capacity for wound healing and to give further antidiabetic benefits. Using Se NPs may also aid in lowering diabetes-related oxidative stress and inflammation, which can hinder the healing of wounds. Therefore, more investigation is needed to understand the rationale behind the combination of Se NPs with PRP for wound healing in diabetic mice. The Se NPs and PRP combination offers the challenges to enhance wound healing in diabetic mice by providing a targeted strategy. By leveraging the strengths of both components, it seeks to overcome the limitations of existing therapies. Platelet-rich plasma holds immense promise for its potential in wound healing and antidiabetic effects. However, its limitations, such as instability and limited targeting capabilities, could hinder its effectiveness. This is where selenium nanoparticles come into play. Se NPs are brimming with antioxidant activity. In diabetic conditions, where oxidative stress runs rampant, these antioxidants can scavenge harmful free radicals, protecting cells and promoting tissue repair. Unlike PRP, Se NPs can be tailored with specific ligands that act like Global Positioning System (GPS), guiding them towards specific cell types or tissues involved in wound healing. This targeted approach can maximize their therapeutic effect while minimizing off-target interactions. This study delves into the enigmatic mechanisms by which Se NPs and PRP work together to orchestrate accelerated wound healing in diabetic conditions. By elucidating these precise interactions, the research offers deeper insights into their combined therapeutic potential, paving the way for more effective and targeted treatments for diabetic wounds.

## Results

**Figure 1:**
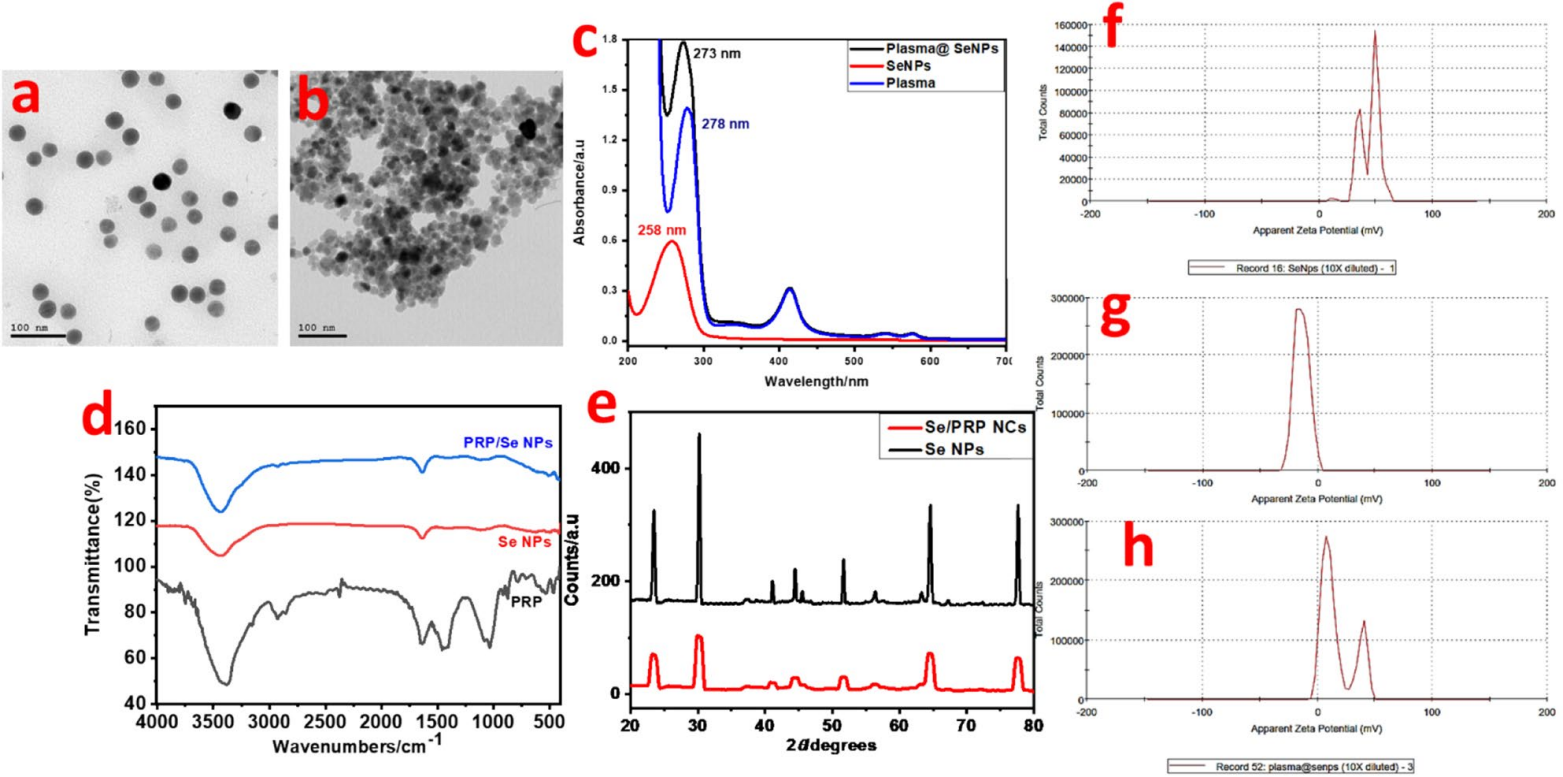
TEM image of the prepared Se NPs and Se/PRP NCs (**a**,**b**). UV–VIS absorption spectra of Se NPs, PRP, and PRP/Se NPs combination (**c**). FRIT spectra of Se NPs, PRP, and PRP/Se NPs combination (**d**). XRD patterns of the prepared Se NPs and Se/PRP NCs (**e**). Zeta Potential Distribution of Se NPs, PRP, and PRP/Se NPs combination (**f**–**h**), respectively.

The transmission electron microscopy (TEM) analysis depicted the spherical shape of the Selenium nanoparticles (Se NPs) with an average diameter of 31.6 ± 1.3 nm (Fig. 1a). Upon the reaction of sodium selenite with ascorbic acid, the selenium underwent reduction to elemental selenium (Se^0^), leading to a colour change from colourless to orange, indicating the occurrence of the reduction reaction and the formation of Se NPs. Se/PRP NCs show slight increase in the particle size to 35 ± 0.6 nm (Fig. 1b). The change in colour of the Se NPs dispersion was further highlighted in the UV–visible absorption spectrum (Fig. 1c), where the maximum absorbance was observed at 258 nm and the combination of Se NPs with PRP showed a shift to longer wavelength by to 273 nm where PRP showed maximum absorbance at 287 nm and 412 nm. The Fourier-transform infrared (FTIR) spectrum of Se NPs (Fig. 1d) exhibited distinct peaks at specific wavenumbers: 3438 cm^−1^ (OH in H_2_O), 2034 cm^−1^ (CN in polyvinylpyrrolidone (PVP)), 1635 cm^−1^ (C = O in PVP), 1116 cm^−1^ (amide in PVP), and 690 cm^−1^ (Se-O). These peaks provided valuable information about the functional groups present in the Se NPs. The purified PRP were characterized using a FTIR spectrophotometer. The hydroxyl group was absorbed as strong intensities between 3000 to 3500 cm^−1^ and ether signals (C–O–C) in sugar units were observed at 2800 to 2950 cm^−1^. A further band at 1050 to 1150 cm^−1^ was due to the aliphatic hydrogen^35^. The combination between Se NPs and PRP showed new peaks at 2800-2950 cm^−1^ of the C–O–C in sugar units of PRP to the FTIR spectrum of Se NPs (Fig. 1d). The X-ray diffraction (XRD) pattern of the Se NPs (Fig. 1e) displayed characteristic diffraction peaks at angles of 23.63º, 30.04º, 41.23º, 44.46º, 45.71º, 52.01º, and 56.49º. These peaks corresponded to the (100), (101), (110), (102), (111), (200), (201), and (003) lattice planes of hexagonal Se, aligning well with the characteristic peaks in the standard card (PDF65-1876). Se/PRP NCs do not show changes in the XRD patterns. XRD is not typically used to analyse PRP due to its limitations in characterizing biological samples. PRP is a complex mixture of various components, including platelets, white blood cells, plasma proteins, and lipids. These components have varying sizes, shapes, and internal structures, making it difficult to obtain clear and interpretable XRD patterns. Se nanosuspension showed water solubility with zeta potential + 44 mV which is a stable suspension system (Fig. 1f). PRP showed zeta potential − 14 mV (Fig. 1g) where the combination between Se NPs and PRP showed + 16.8 mV (Fig. 1h). The zeta potential of + 16.8 mV for the Se NPs-PRP combination suggests limited stability according to Colorado State University's criteria and that may be due to the surface coating of Se NPs can significantly impact their zeta potential. The proteins and other components in PRP can interact with the Se NPs and influence their surface charge. This interaction could lead to a decrease in zeta potential if the interaction is mainly through negatively charged groups. The pH and ionic strength of the suspending medium can also affect the zeta potential. Lower pH or higher ionic strength can generally reduce the magnitude of the zeta potential^36^.

### General observation of wound closure

**Figure 2:**
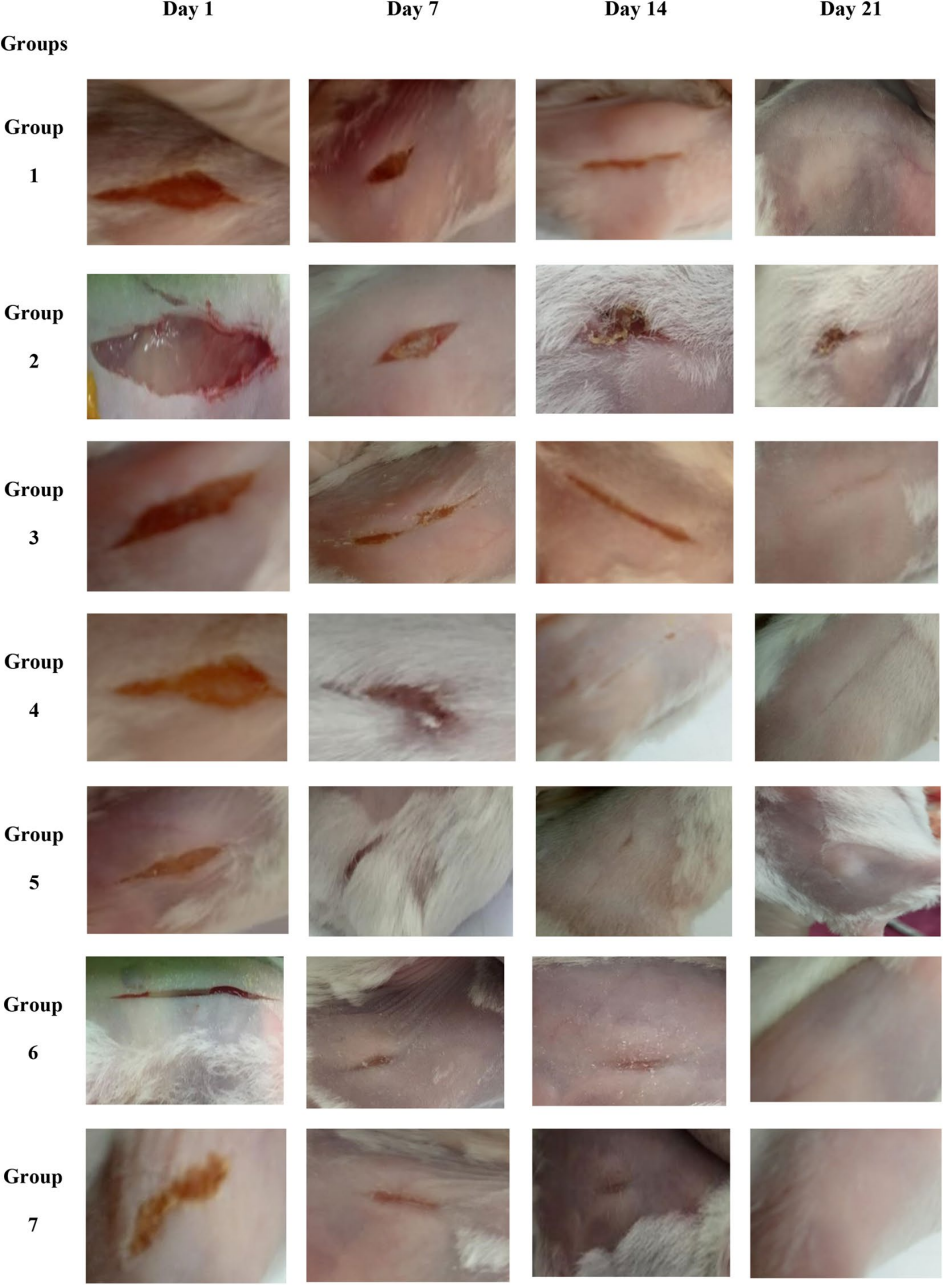
Representative images showing morphological changes of different experimental wound healing groups (incisional wounds on the dorsal surface in mice); a representation of follow-up during the inflammatory phase, reepithelialization and complete healing.

**Figure 3:**
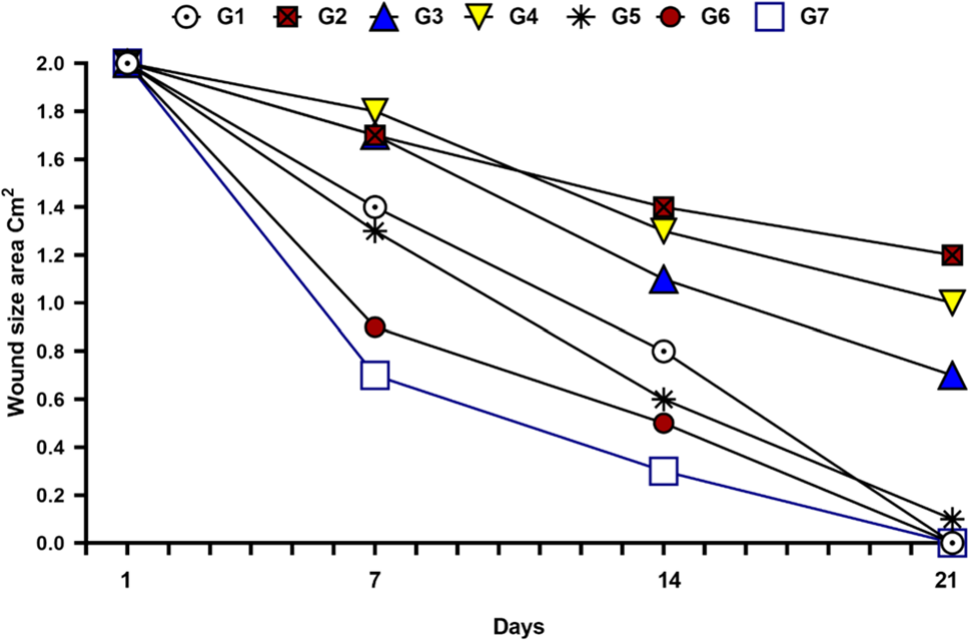
The wound area concerning different days (the rate of wound closure over time).

To examine the therapeutic potential of Se NPs-PRP in diabetic wound healing, an alloxan-induced diabetic mouse model was used to generate type 2 diabetes in mice. The induction efficiency of diabetes with a single dose of alloxan monohydrate (150 mg/kg) was 100% and the survival rate was 100% during the experiment. After surgical wounding of all mice, wound closure was monitored and photo-documented (Fig. 2). Throughout the 21-day treatment period, the mice exhibited no negative effects, as well as none of the experimental groups experienced any appreciable changes in body weight. There was no bleeding or disturbance of the newly formed granulation tissue when all scaffolds were removed. The findings showed that in groups 1, 3, 4, 5, 6, and 7 there was not any clinical or gross sign of intercurrences in wound healing, such as edema, erythema, or suppuration. Groups 5, 6, and 7 showed accelerated wound closure onset. In group 2, the healing process was generally prolonged, incomplete, and uncoordinated until day 21st. Group 3 and 4 showed a slight improvement in healing rate with minor scaring. Figure 3 shows the wound area concerning different days (the rate of wound closure over time).

### The Bates–Jensen wound assessment tool (BWAT)

**Figure 4:**
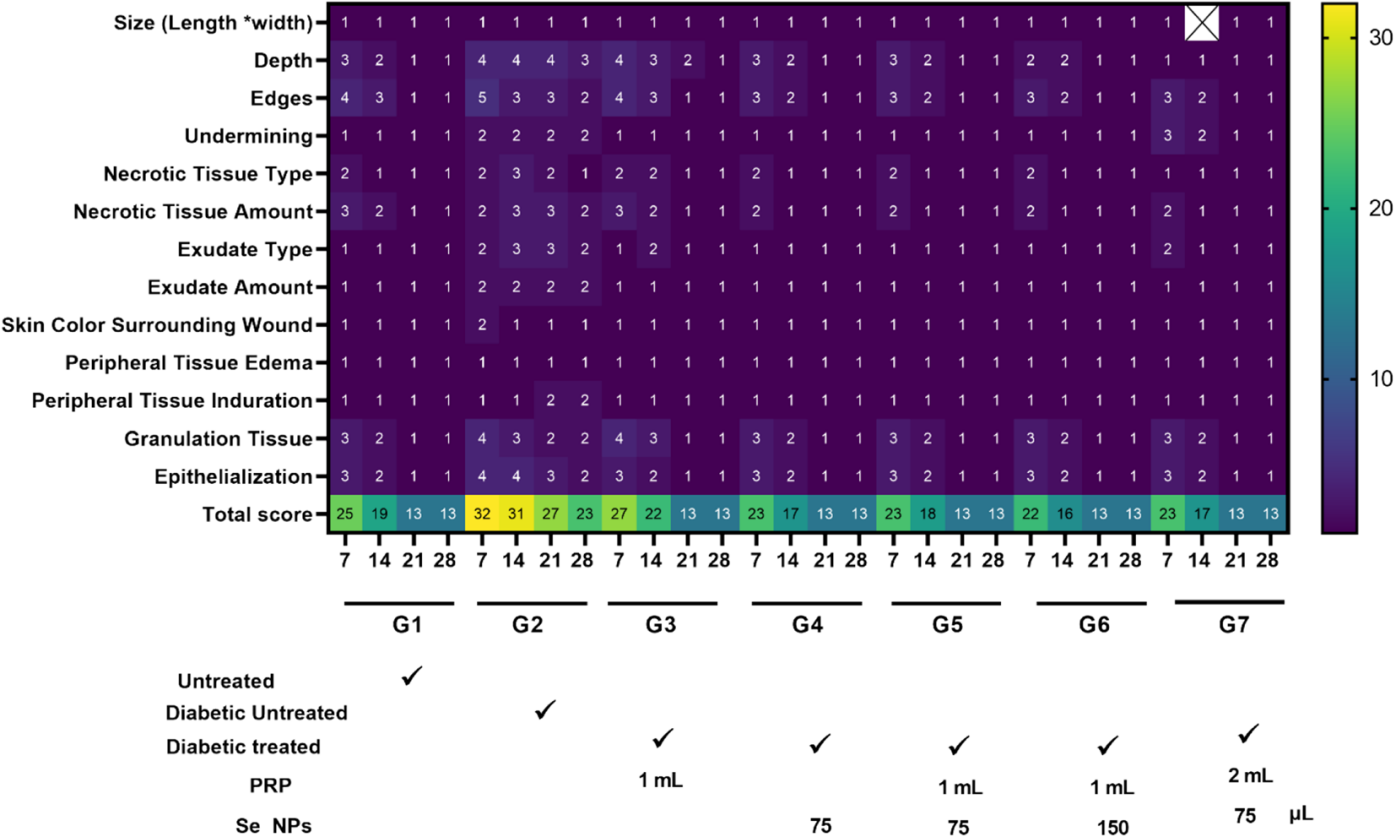
Heat Map representing all wound criteria and scoring of Bates–Jensen Wound Assessment Tool in groups 1:7 group 1 normal untreated mice, group 2; diabetic untreated mice, group 3; diabetic mice treated with PRP, group 4; diabetic mice treated with Se NPs, group 5; diabetic mice treated with Se NPs + PRP (1:1), group 6 Diabetic mice treated with Se NPs + PRP (2:1), group 7; diabetic mice treated with Se NPs + PRP (1:2).

BWAT states that wound regeneration is represented by total scoring less than or equal to 13 while total scoring greater than this value means wound deterioration. To calculate this total score, wound criteria elements are summed up. Each wound criterion is assessed by numbers from 1 to 5. Number 1 refers to improvement toward closure while 5 implies impaired wound healing. From the findings in Fig. 4, cumulative BWAT score values ranged from 13 to 32. Group 2 had higher total scores than the other groups which indicates more severe wound status. All the groups scored 13 on day 21 expect group 2 which scored 27. Until day 28, group 2 scored 23 which means impaired healing process. Heat Map representing all wound criteria and scoring of Bates-Jensen Wound Assessment Tool in groups 1:7 group 1 normal untreated mice, group 2; diabetic untreated mice, group 3; diabetic mice treated with PRP, group 4; diabetic mice treated with Se NPs, group 5; diabetic mice treated with Se NPs + PRP (1:1), group 6 Diabetic mice treated with Se NPs + PRP (2:1), group 7; diabetic mice treated with Se NPs + PRP (1:2).

### Histological examination

**Figure 5:**
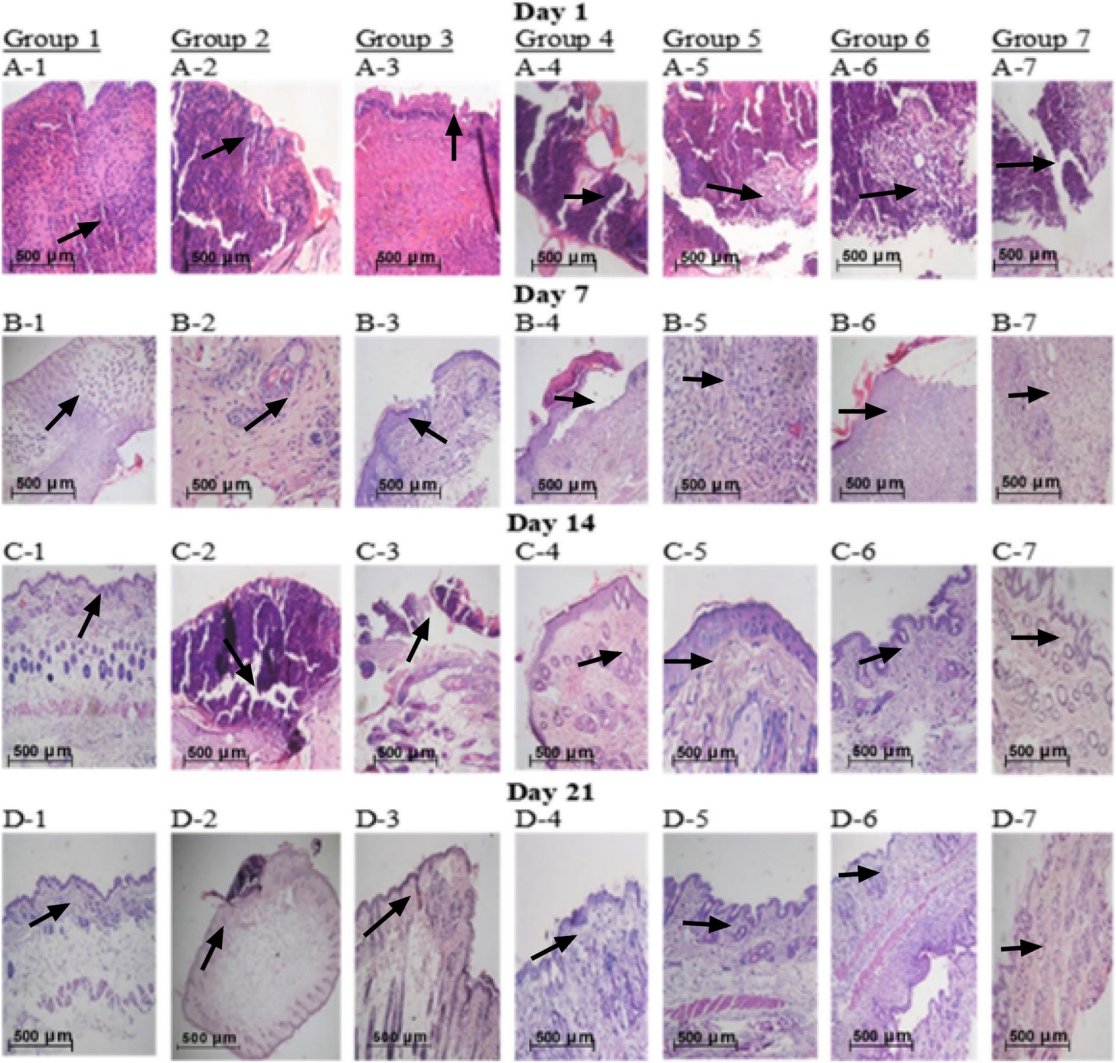
Representative images of H&E-stained histopathological wound sections on days 1,7,14 and 21 post-surgery. (**A**-**1**) acute inflammatory response × 10. (**B**-**1)** full matured granulation tissue. × 10. (**C**-**1**) complete reepithlization. (**D**-**1**) unremarkable skin (completely healed/normal skin) × 10, (**A**-**2**) acute inflammatory response × 10. (**B**-**2**) acute inflammatory reaction × 40. (**C**-**2**) No granulation with a moderate degree of inflammation × 20. (**D**-**2**) no granulation × 10. (**A**-**3**) acute inflammatory response × 10. (**B**-**3**) focal surface denaturation × 20. .(**C**-**3**) ulceration and inflammation × 10. (**D**-**3**) unremarkable skin. (**A**-**4**) acute inflammatory response × 10. (**B**-**4**) acute inflammatory reaction × 10. (**C**-**4**) inflamed granulation tissue. (**D**-**4**) unremarkable skin. (**A**-**5**) acute inflammatory response × 10. (**B**-**5**) granulation tissue with a mild degree of inflammation. (**C**-**5**) reepithlization. (**D**-**5**) unremarkable skin. (**A**-**6**) acute inflammatory response × 10. (**B**-**6**) extensive granulation tissue formation × 10. (**C**-**6**) reepithlization. (**D**-**6**) unremarkable skin (completely healed skin). (**A**-**7**) acute inflammatory response × 10. (**B**-**7**) acute inflammatory reaction × 10. (**C**-**7**) reepithlization. (**D**-**7**) unremarkable skin. The histological scores (Table 2) revealed incomplete wound healing in group 2, and moderate wound healing in groups 3 and 4. Groups 5,6 and 7 revealed a fast wound healing rate approximately equal to the normal rate of group 1(normal mice).

Histological examination of the samples treated with Se NPs, PRP, Se NPs/PRP, and control group was examined using H&E illustrated in Fig. 5. On day 1 all the groups showed a cute inflammatory response as shown in Fig. 5 (a-1), (a-2), (a-3), (a-4), (a-5), (a-6), and (a-7). By day 7, groups 1, 5, 6, and 7 showed abundant granulation tissue with thick layers. Whereas group 3 and 4 exhibited less granulation with moderate inflammation. Group 2 showed congested blood vessels, extravasated RBCs and mononuclear inflammatory cell infiltration. By day 14, groups 1, 5, 6, and 7 exhibited significantly faster epithelial regeneration. Group 3 and 4 exhibited decent maturation degree and newly formed epidermal rete ridges. Until day 21, group 2 demonstrated defective healing and poor epithelialization. The histological scores revealed incomplete wound healing in group 2, and moderate wound healing in groups 3 and 4. Groups 5, 6, and 7 revealed a fast wound healing rate approximately equal to the normal rate of group 1 (normal mice) as shown in Table 1. From these histological results, Se NPs-PRP is shown to enhance wound granulation tissue formation and epithelialization to accelerate wound closure.

**Table 1:** Histopathological assessment of wound healing parameters.

Groups	Collagen formation	Fibroblast proliferation	Angiogenisis	Epithelization
Group 1	+ + +	+ + +	+ + +	+ + +
Group 2	+	+	+	+
Group 3	+ +	+ +	+ +	+ +
Group 4	+ +	+ +	+ +	+ +
Group 5	+ + +	+ + +	+ + +	+ + +
Group 6	+ + +	+ + +	+ + +	+ + +
Group 7	+ + +	+ + +	+ + +	+ + +

+  +  + HIGH presence of extracellular matrix, +  + MODERATE presence of extracellular matrix and + DISCRETE incomplete presence of extracellular matrix.

### Histochemical observations

#### Hydroxyproline

**Figure 6:**
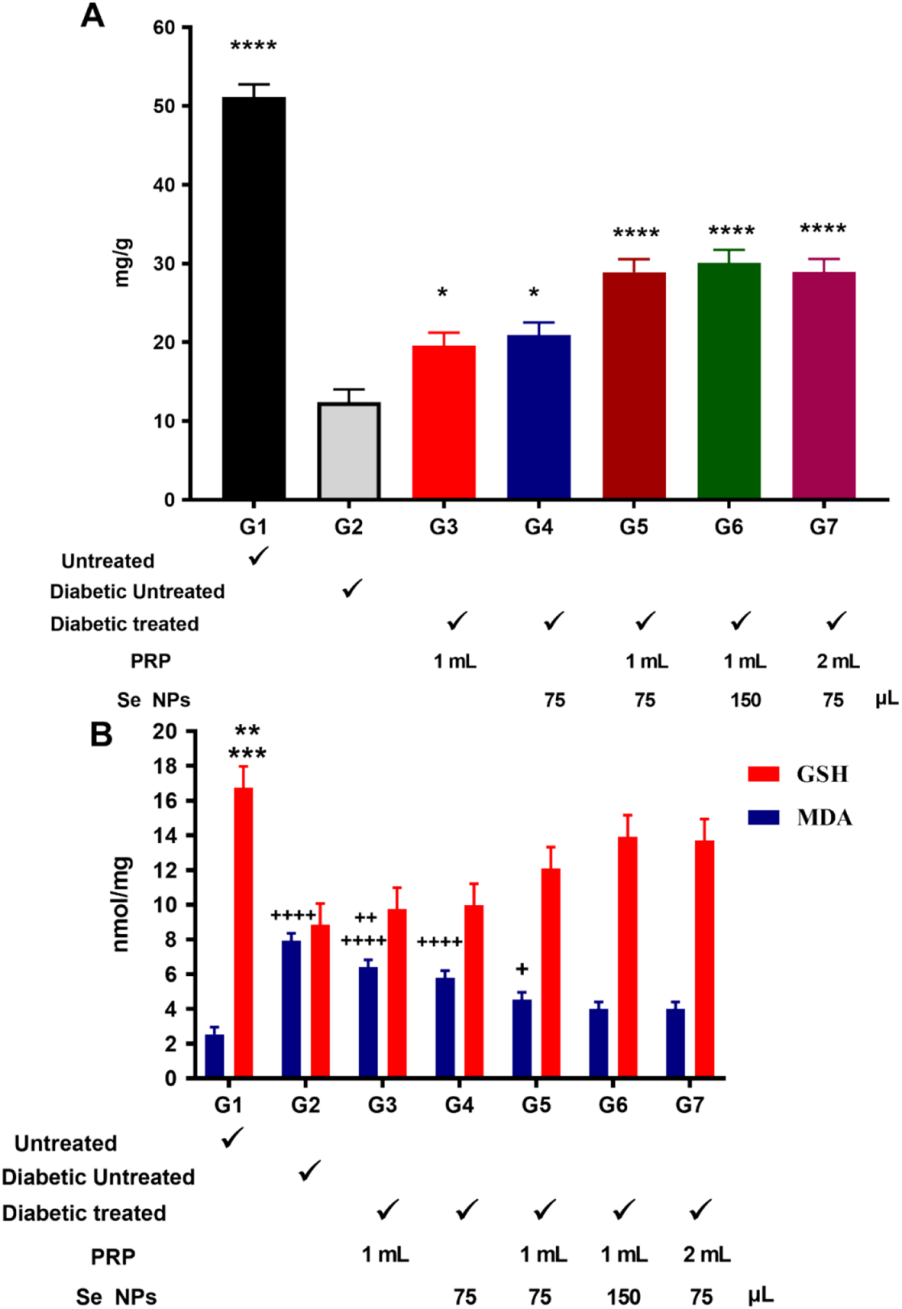
Hydroxyproline content, oxidative stress and antioxidant activity. (**A**) Hydroxyproline content as measured in mg/g. (**B**) GSH and MDA measurement in nmol/mg and are shown as the means and SEM. Statistical significance of differences was analyzed by one-way ANOVA with Tukey’s posttest, For hydroxyproline: *p < 0.05, G2 versus G3 & G4; G4 versus G5; G4 versus G7; **p < 0.01, G3 versus G5 & G7; G4 versus G6; ***p < 0.001, G3 versus G6; ****p < 0.0001 G1 versus G2, G3, G4, G5, G6, G7;G2 versus G5, G6& G7. For GSH: **p < 0.01, G1 versus G3 and G4; ***p < 0.001, G1 versus G2. For MDA: ^**+**^p < 0.05, G1 versus G5; G2 versus G4; G3 versus G5; G4 versus G6 & G7; ^**++**^p < 0.01, G3 versus G6 & G7; ^**++++**^p < 0.0001 G1 versus G2, G3, G4 ; G2 versus G5, G6, G7.

Healing tissues synthesize collagen, which is a component of growing cell. Hydroxyproline acts as marker of collagen that’s why we evaluated hydroxyproline content in skin tissue. Concentration of hydroxyproline is a reflection and quantification of the collagen concentration. Higher hydroxyproline concentration implies faster wound healing rate. Figure 6 gives an overview of the findings. Hydroxyproline contents in groups 1 to 7 were 51.1, 12.39, 19.61, 20.90, 28.94, 30.10, and 28.96 mg/g, respectively (Figure 6). Among the diabetic groups, the maximum levels of hydroxyproline were documented in group 6 (30.10 mg/g) which implies more collagen deposition and fast healing rate. While there was a slight difference in hydroxyproline level in group 5 and group 7 (28.94 mg/g and 28.96 mg/g respectively). Hydroxyproline level in group 2 decreased by 4.12 times in comparison with group 1 (normal mice). Hydroxyproline levels in group 3 and 4 were relatively elevated but still less than groups 5, 6, and 7.

#### Oxidative stress and antioxidant activity

Systemic oxidative stress and cellular stress response in all the experimental groups were evaluated and represented by Malondialdehyde** (**MDA). Antioxidant Enzymes Glutathione (GSH) level gives a reflection of the antioxidant potential of Se NPs-PRP (Fig. 6).

#### MDA

The lipid peroxidation status of MDA value of groups 2, 3, 4, 5, 6, and 7 was seen higher than that of group 1(normal control). While assessing the oxidative stress status, an increase in the average MDA in groups 3 and 4 was revealed compared to groups 5, 6, and 7. Group 6 and 7 showed the same MDA level. Group 2 showed the greatest value (7.94 nmol/mg).

#### GSH

Antioxidant activity of Se NPs-PRP were given in Fig. 6. GSH level was the greatest in group 6 (13.93 nmol/mg). The value of GSH levels in group 2 exhibited a significant decrease (to 52.86% of control levels). Groups 3 and 4 showed similar values (9.76 and 9.98 nmol/mg). However, a remarkable increase was noticed in groups 5 and 7 comparing to groups 2, 3, and 4. Findings of MDA and GSH validates the potent wound healing activity of Se NPs-PRP. As it reduced oxidative stress, and increased glutathione levels.

## Discussion

This in vivo study was carried out to determine the wound healing activity of Se NPs-PRP in diabetic mice. The speed of wound healing was assessed according to the given treatment. On the 7th day, the wound area ratio was significantly small in groups 1, 5, 6, and 7 implying significant wound healing activities. Groups 3 and 4 showed moderate healing activity. Group 2 showed the lowest healing activity. On the 14th day, groups 1, 3, 4, 5, 6, and 7 appeared non-inflamed with epithelial tissues attached completely without signs of infection. Group 2 had inflamed wounds with evidence of infection. On the 21st day, the wounds of all groups were almost completely closed except group 2. Groups 5, 6, and 7 showed faster hair regrowth rate. By the 28th day, group 2 showed delayed wound closure and impaired wound contraction compared to all other groups. In Group 3, moderate healing activity is explained by PRP treatment. PRP accelerates the healing of diabetic wounds through several potential mechanisms. Their increased concentration of growth factors lowers inflammation. They also release antimicrobial peptides and serve as antibiosis agent^37^. Moreover, PRP potential antidiabetic effect though stimulated islet cell regeneration has been reported^38^. In Group 4, moderate healing activity is explained by Se NPs treatment. Se NPs treatment accelerates wound healing through several mechanisms. Their antihyperglycemic activity has been demonstrated in streptozotocin-induced diabetic rats^39^.

Se NPs with their small size and large surface area have high potential for scavenging free radicals. The oxidative stress is recognized to greatly affect the regenerative potential and cause delayed wound healing^40^. Moreover, Se NPS antimicrobial effect through disruption of bacterial cell wall is suggested to tackle bacterial resistance encountered in diabetic wounds^41^. In Groups 5, 6, and 7, the wound healing activity was significant. This implied significant synergistic effect of the Se NPs-PRP combination. Group 6 showed the highest wound healing activity with their specific ratio values.

By the end of the experiment, wound healing was observed in all histological specimens of all study groups except group 2. On the 1st day, all specimens showed acute inflammatory response as a defence mechanism against the occurred injury. This inflammation is characterized by accumulation of leukocytes within the connective tissue (injury site). On the 7th day, a layer of epithelium started to appear on the granulation tissue of all the groups except group 2. This layer is an indication of the proliferation of basal cells around the wound, and migration to the midpoint of the wound. Group 2 showed vast amounts of inflammatory cell infiltration with no granulation until week 2. Epithelialization was observed earlier in groups 1, 5, 6, and 7. At the end of the experiment period, all skin wounds healed both macroscopically and microscopically and re-epithelialized completely except group 2.

Histological scores revealed incomplete wound healing in group 2, moderate wound healing in groups 3and 4 and fast wound healing in groups 5, 6, and 7, close to that, of group 1 (normal mice). The primary indicators of wound healing include collagen formation, fibroblast proliferation, angiogenesis, and epithelialization. Group 2 showed poor collagen formation, fibroblast proliferation, angiogenesis, and epithelialization. Groups 3 and 4 showed moderate collagen formation, fibroblasts proliferation, angiogenesis, and epithelialization. Groups 5, 6, and 7 showed greater collagen formation, fibroblast proliferation, angiogenesis, and epithelialization.

The proposed mechanisms for microscopic wound healing activity are brought by PRP and Se NPs. PRP growth factors produce fibroblasts and endothelial cells which the body uses to subdue inflammation and promote healing. PRP promotes angiogenesis of wound tissue by increasing local vessel intensity and its associated (VEGF) and insulin-like growth factor 1. Furthermore, PRP promotes wound contraction and stabilizes collagen arrangement^42^. Se NPs treatment decreases apoptosis and promotes wound healing through their antioxidant and anti-inflammatory effects^43^.

These findings were in good accordance with Webster et al., who reported mild improvement of dermal fibroblast metabolic activity with adding Se NPs to electrospun silk Scaffold^44^. Also, Xu et al. indicated accelerated wound healing potential of Se NPs by reducing inflammation, enhancing granulation tissue formation, and angiogenesis. In addition to the effect of Se NPs in accelerating wound healing process we added PRP which boost the activity of Se NPs. PRP regulate the production of inflammatory cytokines interleukin-17A and interleukin-1β and increased the local vessel intensity and re-epithelialization. Besides the enhanced secretion of growth factors such as (VEGF) and (IGF-1). PRP also increases the migration and proliferation of primary cultured ESCs which accompanied by the differentiation of ESCs into adult cells following the changes of CD49f. and keratin 10 and keratin 14. PRP promotes collagen deposition and effectively reduce healing time^42^.

A healing tissue synthesizes collagen, which is a constituent of growing cells. Concentration of hydroxyproline indicates faster wound healing. Hydroxyproline levels in group 2 decreased in comparison with other groups which reflected poor wound healing. Hydroxyproline levels in groups 3 and 4 increased relatively but less than the increase in groups 5, 6, and 7. This shows the therapeutic effects of PRP and Se NPs on wound healing activity reflected in higher hydroxyproline levels. The higher levels in groups 5, 6, and 7 suggest the synergistic therapeutic effect when combined. These results are in line with Zarei et al. who reported significant increase in hydroxyproline levels with chitosan/Nano Selenium Biofilm in rats^45^. Also, Jiang indicated remarkable increase of hydroxyproline levels with chitosan‐ZnO/Selenium nanoparticles scaffold^46^. Another study by Nafiu et al. demonstrated that Se and Papaya extract increased the healing potency by up-regulation of antioxidant enzymes and cyclooxygenase specific inhibition^47^.

The increase in the lipid peroxidation status and the decline in the antioxidant defence mechanisms are the main factors in the development of complications in diabetes mellitus. Excess of lipid peroxidation and reactive oxygen species (ROS) formation damage normal cells and delay wound healing^48^. For better understanding, lipoperoxidation results from the interaction of hydroxyl radicals with unsaturated fatty acids in cell membranes, leading to the formation of lipid peroxides, hydroxide radicals, and MDA^49,50^. In this study the oxidative stress status was assessed by measuring MDA and the antioxidant status by measuring GSH which protects against accumulation of free radicals in the cell^51^. Reduced GSH levels contributes to delay wound healing process^52^. Lipid peroxidation status of MDA of all groups were higher than group 1 (normal mice). Group 2 scored the highest value due to high oxidative stress. There was decrease in the average MDA in groups 3 and 4. More decrease in MDA levels in Groups 5, 6, and 7 suggesting less oxidative stress due to the therapeutic effect of Se NPs-PRP. GSH is a protective enzyme against free radical formation in tissues and it decreases in diabetic mice due to inactivation caused by generated ROS. Group 6 showed the highest GSH activity while group 2 represented the lowest GSH value (8.85 nmol/mg). Groups 3 and 4 showed similar results with values 9.76 and 9.98 nmol/mg. Significant increase was noticed in groups 5 and 7 compared to groups 2, 3, and 4. Se NPs reversed the activation of this enzymatic antioxidant, which might be due to decreased oxidative stress as evidence by decreased MDA levels too. Also, Se NPs are characterized by small size and their high surface area. This small size allows more Se atoms to scavenge a lot of free radicals. Thus, Se NPs have great scavenging activity, and boost endogenous antioxidant proteins^53^. A similar study investigated the ability of Se NPs to quench ROS and demonstrated that Se NPs inhibited lipid peroxidation in renal tissues, reflecting its ability to protect the integrity of the cell membrane^54^. It was also reported that Se NPs could elevate GSH and mRNA expression of its based enzymes (GPx and GR) in addition to (SOD) and catalase (CAT).

Based on all the obtained results, it can be concluded that Se NPs-PRP may have a potential in wound healing activity. As shown morphologically, the wound closure in diabetic mice groups treated with Se NPs-PRP (with different ratios) was very close to normal nondiabetic mice. The histological study revealed that, Se NPs could elevate the rate of proliferation, revascularization, collagen deposition and remodelling at wound sites. This agreed with the histochemical investigation of hydroxyproline which showed raised levels. Decreased MDA levels could be due to reduced oxidative stress. Increased activity of GSH may indicate the antioxidant nature of both Se NPs and PRP against oxidative stress which may have a contributory role in fasten wound healing process.

Platelet-containing plasma (PRP) has shown potential for wound healing, but it faces many challenges. First, the lack of standardization in PRP preparation leads to variation in the number of platelets that promote platelet growth, affecting the efficacy and complexity of treatment Second, although PRP provides shorter wound healing times however, the long-term benefits remain unclear, and studies suggest that treatment effects may diminish over time. In addition, PRP injections can cause side effects such as pain, inflammation, and delayed healing especially in individuals with certain underlying conditions Apart from that, the mechanisms of PRP for scarring are not understood is effectively treated is well understood, and more studies are needed to clarify the role of various tumour factors and cellular properties. Most clinical studies of PRP in wound healing are limited or lack rigorous methodologies, emphasizing the need for large, well-controlled trials to provide conclusive evidence of PRP efficacy emphasize efficiency and safety.

## Conclusion

The application of nanoparticles in the treatment of diabetic wound healing is proving to be a promising and effective tool in treatment and care. Nanomaterials can stimulate several cellular and molecular processes that help with wound microenvironment through their antimicrobial, anti-inflammatory and angiogenic effect. The unique properties of selenium nanoparticles make them the therapeutic of choice in diabetic wound healing with their superior antioxidant and antimicrobial effect. Adjuvant PRP therapy can further speed the healing process in diabetic wounds with their increased concentration of growth factors. They jump start healing from inflammatory to proliferative stage. PRP triggers the body’s natural healing properties, to repair and regenerate cells and tissues. The prospects of Se NPs and adjuvant PRP would be the breakthrough therapeutic in tackling the complexity of diabetic wound healing. A synergistic therapeutic effect on diabetic wound healing was demonstrated. Se NPs-PRP showed promising potential in modulating wound healing in diabetic mice. These results would have future therapeutic implications for diabetic wounds in humans. It could tackle the complexity of (DFU) and lower the incidence of lower limb amputations.

## Materials and methods

### Preparation of Se NPs

Se NPs were produced by simple wet chemical method in which 0.1g of polyvinylpyrrolidone (PVP) was dissolved in 40 mL of deionized water with continuous stirring. 0.6 g of ascorbic acid was added to the prepared solution while stirring for 5 min. Then 1mL of 0.96g/mL of cetyltrimethylammonium chloride (CTAC) solution was added. After 5 min of stirring 1mL of 0.25M of sodium selenite (SS) solution was added drop-wisely until the solution changed from colorless to orange^55,56^.

### Preparation of PRP

Healthy group of mice was used for collecting the whole blood for PRP preparation. Whole blood (WB) by venipuncture was obtained and placed in acid citrate dextrose (ACD) tubes at room temperature then centrifuged using a soft spin. The resultant supernatant plasma containing platelets was transferred into another sterile tube (without anticoagulant) then centrifuged at a higher speed (hard) spin. The platelet-poor plasma (PPP) in upper 2/3rd of the tube was removed. The remaining PRP was in the lower 1/3rd at the bottom of the tube.

### Preparation and characterization of Se NPs-PRP

4 mL of plasma was dissolved in 4 ml of deionized water and was mixed with 75 microns of the prepared nanoparticles. UV–VIS absorption spectra were obtained on Cary series UV–Vis-NIR, Australia. Transmission Electron Microscopy (TEM) images were performed on Talos F200i high resolution transmission electron microscope (Thermo-Fisher) at an accelerating voltage of 200 kV. X-ray diffraction (XRD) measurements were conducted using a Philips PW1710 X-ray diffractometer with Cu Ka radiation (k = 1.54186 A˚). The XRD patterns were recorded within the 20° to 70°2Ɵ range with a step size of 0.020°2Ɵ and a collection time of 10 s per step. FT-IR spectra were recorded using a Nicolet 6700 infrared spectrophotometer to identify specific functional groups present on the surface. The charge density measurements were carried out via malvern panalytical zetasizer nano zs.

### In vivo study

#### Animal ethical approval

All animal experiments were performed in accordance with the ethical approval number CU I F 42 21 from Institutional animal care and use committee (CU-IACUC) Cairo University. This study is reported in accordance with ARRIVE guidelines^57^. All experiments were performed in accordance with relevant guidelines and regulations in the National cancer institute, Cairo University. The animals were housed in polypropylene cages and allowed free access to food and water. The animals were acclimatized to standard laboratory environmental conditions, dark and light cycles (12:12 h) for 28 days.

### Induction of diabetes

After 16 h of fasting, 150 mg/kg alloxan monohydrate was intraperitoneally administrated^32^. Following an alloxan injection, animals frequently exhibit increased thirst and polyuria (excessive pee) due to insulin insufficiency and hyperglycemia. As a result, the consumption of glucose water was monitored following an alloxan injection to determine how the injection affects blood sugar levels. Intake of glucose water is frequently tracked following an alloxan injection to evaluate how the injection affects blood sugar levels. Following surgery, housing arrangements for experimental mice usually entail giving them a clean, safety, cosy space with access to food and drink, as well as keeping an eye on their recuperation and general wellbeing. After five 5 days with access to food and water, the mice were screened for hyperglycemia. Mice with blood glucose greater than 200 mg/dl were considered diabetic^58^. Blood glucose was monitored using a glucometer.

### Experimental design

Forty-nine male mice of the BALB/c strain aged two months with uniform weight (23 ± 0.5g) were divided into seven groups (n = 7) (Table 2). Group 1 represents untreated mice that received only food and water for 28 days. The other six groups (2–7) represent diabetic mice.

**Table 2:** Analysis of the studied groups within the present work.

Mice groups	Description
Group 1	Untreated mice (negative control)
Group 2	Diabetic untreated mice (positive control)
Group 3	Diabetic mice treated (IP) with 1 mL of PRP (Treatment 1)
Group 4	Diabetic mice treated (IP) with 75 µL Se NPs (Treatment 2)
Group 5	Diabetic mice treated (IP) with 75 µL Se NPs + 1 mL PRP (1:1) (Treatment 3)
Group 6	Diabetic mice treated (IP) with 150 µL Se NPs + 1 mL PRP (2:1) (Treatment 4)
Group 7	Diabetic mice treated (IP) with 75 µL Se NPs + 2 mL PRP (1:2) (Treatment 5)

### Surgical wounding

For making injuries, mice were anesthetized using 5% isoflurane. The dorsal portion of the body was shaved, and sterilized. Using a scalpel, make a cut line on each mouse (2 cm long-incision) was made. The incision was made paravertebral and perpendicular to the skin cleavage line. All wounded mice were photo-documented to evaluate the healing rate clinically. Following surgical procedures, housing arrangements for experimental mice usually entail giving them a clean, safety, adequate space with access to food and drink, as well as keeping an eye on their recuperation and general wellbeing. The analgesic (Ketoprofen) was given intramuscularly (IM) after surgery to alleviate pain till the symptoms were resolved.

### Morphological evaluation

Photo-documentation of wound closure in all groups over 28 days was carried out. Also, Bates-Jensen Wound Assessment Tool (BWAT) was used to assess wound closure rate^33^. It is a straightforward validated wound assessment tool that allows for detailed reassessment and monitoring of healing process. This assessment tool includes 13 items such as wound size, type and depth, amount of necrotic tissue, amount of exudate, granulation tissue formation and epithelialization which documents details of the wound to improve treatment planning and re-assessment. Scores range from 1 to 5. Score 1 indicates the best healing situations while score 5 indicates impaired healing or wound deterioration^53^.

### Histological evaluation

Histopathology of wounds is an essential approach to monitor healing progress, assessing morphological changes in each phase and help with diagnosis and treatment of impaired wounds^59–61^. Clinically, taking the biopsy at the edge of the wound makes the comparison between the ulcerated area and the surrounding skin easier. However, in the laboratory setting a biopsy for histopathological analysis should encompass the whole wound^62–64^. Skin biopsies were taken on days 1, 7, 14, and 21 post-surgeries. Skin samples were placed immediately into specific solutions such as 10% buffered formaldehyde and subjected to many steps of histological processing, including embedding, sectioning (4 μm formalin fixed paraffin embedded sections) and staining with hematoxylin and eosin (H&E)^65^.

### Histochemical evaluation

#### Evaluation of collagen content through hydroxyproline:

Collagen breakdown releases free hydroxyproline and its peptides. That’s why hydroxyproline is used as a biochemical marker during wound healing for evaluation of tissue collagen content. It is also used as an indicator of collagen turnover after wound-healing. Increased hydroxyproline content in granulation tissue reflects increased collagen turnover, which refers to higher maturation and proliferation of collagen during wound closure^66,67^.

#### Hydroxyproline

To evaluate hydroxyproline, 4-mm-diameter skin biopsies were obtained after the 14th post wound day and dried in a hot-air oven at 60–70 °C. Then, all the tissue samples were hydrolyzed with 6 N HCl for 3 h at 130 °C. The hydrolyzed tissue samples were adjusted to pH 7.0 and subjected to chloramine-T oxidation. The colored adduct product formed with Ehrlich reagent at 60 °C was read at 557 nm. According to Woessner, Standard hydroxyproline was run and values reported as mg/g dry weight of tissue^68^.

#### Oxidative stress tests

The parameters of oxidative stress include Lipid peroxidation products (LPO) and the activity of antioxidant enzymes Glutathione (GSH). Their levels were assessed to follow up wound healing activity and the potential antioxidant effect of the nano-combination Se NPs-PRP^40^.

### Lipid peroxidation

#### Malondialdehyde (MDA)

MDA is a major indicator of oxidative stress as it used for screening and monitoring lipid peroxidation induced by ROS. This assay was carried out using ab233471 Lipid Peroxidation (MDA) Assay Kit (Colorimetric). Absorbance increase was monitored at 695 nm and MDA concentration was expressed as nmol/mg protein.

#### Reduced glutathione (GSH)

GSH was measured via fluorometric method^69^.

### Statistical analysis

Statistical analysis was performed using Graphpad prism version 9, one-way ANOVA with Tukey’s posttest.

For quantitative variables of triplicate determinations, data were presented as Mean and standard deviation (SD).

## Data Availability

All data generated or analyzed during this study are included in this published article. Additionally, the raw data is available in a public repository at [https://pubmed.ncbi.nlm.nih.gov/33907960/] with 10.1007/s11356-021-14089-w].
